# Rapid Classification and Identification of Chemical Components of *Schisandra Chinensis* by UPLC-Q-TOF/MS Combined with Data Post-Processing

**DOI:** 10.3390/molecules22101778

**Published:** 2017-10-20

**Authors:** Shenshen Yang, Lanlan Shan, Houmin Luo, Xue Sheng, Jun Du, Yubo Li

**Affiliations:** Tianjin State Key Laboratory of Modern Chinese Medicine, School of Traditional Chinese Materia Medica, Tianjin University of Traditional Chinese Medicine, 312 Anshan West Road, Tianjin 300193, China; shanlanlan12@163.com (L.S.); luohoumin2016@126.com(H.L.); m15222839077@163.com(X.S.); wennypluto@163.com(J.D.)

**Keywords:** WuWeiZi(WWZ), UPLC-Q-TOF/MS, characteristic fragments (CFs), neutral losses (NLs)

## Abstract

*Schisandra chinensis* (known in Chinese as WuWeiZi, WWZ) has observable effects such as astringing the lung to stop coughs, arresting sweating, preserving semen and preventing diarrhea. The major components of WWZ include lignans, triterpenoids, organic acids and fatty acids. In this paper, a reliable method for the rapid identification of multiple components in WWZ by their characteristic fragments and neutral losses using UPLC-Q-TOF/MS technology was developed. After review of the literature and some reference experiments, the fragmentation pattern of several compounds were studied and summarized. Then, according to the corresponding characteristic fragments coupled with neutral losses in the positive or negative ion mode produced by different types of substances a rapid identification of target compounds was achieved. Finally, a total of 30 constituents of WWZ were successfully identified, including 15 lignans, nine triterpenoids, three organic acids and three fatty acids. The method established in this study not only provides a comprehensive analysis of the chemical ingredients of WWZ, providing a basis for further phytochemical studies on WWZ but also provides a more efficient way to solve the problem of identification of complex chemical constituents in traditional Chinese medicines.

## 1. Introduction

*Schisandra chinensis* (the dried mature fruits of *Schisandra chinensis* (Turcz.) Baill or *Schisandra sphenanthera Rehd* et Wils) is commonly used as a tonic, sedative and an anti-aging drug in clinical practice, for it has good anti-inflammatory, anti-oxidative, immunomodulatory and anti-hepatic injury effects [1,2].The main components in *Schisandra chinensis* (named WuWeiZi, WWZ) contain lignans, triterpenoids, organic acids and fatty acids, volatile oils, and sugars. Many studies have shown that lignans, which have various biological activities such as antihepatotoxic, antioxidant and detoxificant effects, are the major bioactive constituents of WWZ [3,4,5], able to inhibit hepato-carcinogenesis and enhance human intellectual activity [6]. Triterpenoids show anti-HIV effects and inhibitory activities toward cholesterol biosynthesis [7]. Organic acids show antibiotic pharmacological activities [8], while fatty acids have been associated with anti-inflammatory, immunomodulatory, analgesic and anti-tumour properties [9]. Compared to the single compounds of Western medicine, the efficacy of traditional Chinese medicines (TCMs) is characterized for its integrity and synergy resulting from the various components. However, according to current research results, the qualitative identification of WWZ is mainly concentrated on the lignans [10,11]. Therefore, it is not only necessary to develop multi-component fingerprints by UPLC-Q-TOF/MS, but more importantly to establish a rapid and reliable method for the identification of different types of constituents in WWZ.

Many methods for analysing chemical ingredients of WWZ have been reported, including thin layer chromatography (TLC) [12], high-performance liquid chromatography (HPLC) [13], capillary electrophoresis (CE) [14], high speed countercurrent chromatography (HSCCC) [15], gas chromatography-mass spectrometry (GC/MS) [16], among others. Nowadays, ultra-performance liquid chromatography/time-of-flight mass spectrometry (UPLC-Q-TOF/MS) is widely used [17,18]. With the rapid development of modern analysis technology and data post-processing technology, characteristic fragments filter (CFF) and neutral loss filter (NLF) represent two data post-processing technology methods that can be used for the in-depth study of TCM components [19,20]. Under identical MS conditions, compounds with same or similar parent nuclei could be split into different fragments. Characteristic fragment ions (CFs) refer to the fragments that could be used to infer the cleavage types and the classification of substances, which helps to screen the target components and filter congeners. Moreover, neutral fragments (NFs) lost during cleavage processes also play an important role in screening substances, as they reflect the discrepancy of the *m*/*z* between the parent ion and the fragment ions in high mass-to-charge ratio portions [21,22,23,24,25,26]. Therefore, it is propitious to realize the rapid screening and qualitative analysis of constituents of TCM combined with data post-processing techniques (CFF and NLF) on the basis of UPLC-Q-TOF/MS.

The rapid identification and analysis of the components are helpful to further develop pharmacodynamic material basis and determine the functional mechanism(s) of TCMs. Therefore, analytical methods should be developed to rapidly classify and identify complex TCM components. This paper is mainly based on the UPLC-Q-TOF/MS method. Firstly, the mass spectrometry information in regard to the components of WWZ extract were classified and summarized after a review of the literature and some reference experiments results. According to the above results, it can be speculated that fragmentation regularity exists in the mass spectra of several different constituents of WWZ. Then, the CF and NL rules of lignans, triterpenoids, organic acids and fatty acids were established and generalized. Then, original mass spectral information was obtained and processed by the Masslynx version 4.1 software (Waters, Milford, UK) to detect and align the peaks. Finally, the identities of these compounds were determined on the basis of mass information in combination with different CFs and NLs. In this study, more abundant mass spectrometry information was acquired by scanning in the positive and negative ion mode, and in combination with a characteristic post-processing technique for information integration, several compounds could be classified and identified. To a certain degree, the method provides an effective way for solving the key issue of rapid classification and identification of complex chemical components in TCMs, and also a foundation for controlling the quality of different batches of original herbs.

## 2. Results and Discussion

### 2.1. The Establishment of Data Post-Processing Technology Based on UPLC-Q-TOF/MS

**Figure 1:**
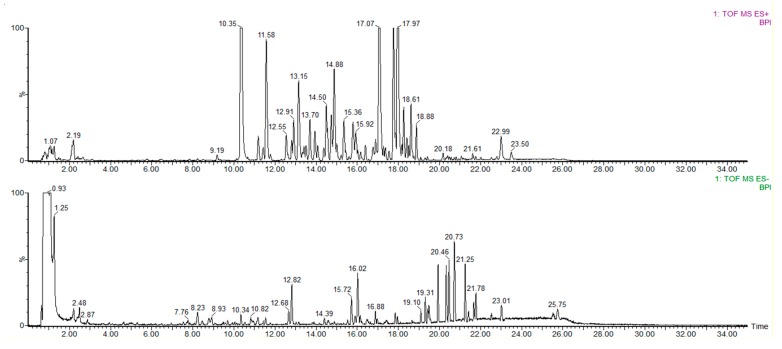
Typical total ion current (BPI) chromatograms of substances in the WWZ extract, under positive and negative ion modes.

In order to obtain further material information of the fingerprint, based on the physical structure and chemical characteristics of components in WWZ; a BEH C18 column was eventually used. Good resolution with high and narrow peaks were obtained at a column temperature of 35 °C, flow rate of 0.4 mL·min^−1^, injection volume of 2 µL and with a mobile phase consisted of (**A**) 0.1% formic acid in water and (**B**) acetonitrile containing 0.1% formic acid. Additionally, the WWZ extract was comprehensively analysed under positive and negative ion modes. Typical BPI chromatograms of the substances in the WWZ extract in both modes are shown in Figure 1.

In this study, WWZ extract was selected for the experiments. Firstly, according to the existing literature, the fragmentation information about the lignans, triterpenoids, organic acids and fatty acids as main components in WWZ was collected. Then, the fragmentation rules of CFs and NLs with regard to the above four types of components were inferred and are listed in Table 1, and the ion information of the constituents could be obtained using the Masslynx software.

Substances can be classified by NLs, that refers to the mass difference between molecular ion peaks and high mass-to-charge ratio fragment peaks, combined with the CFs of other subtypes to determine the type of compound. Finally, a method was established for rapid identification and classification of chemical compositions in WWZ extract by using CFF and NLF. Lignans are generally divided into biphenyl cyclooctene lignans and open-loop lignans. In line with the fragmentation information obtained in mass spectrometry from the literature and the total ion flow mass spectrometry information, biphenyl cyclooctene lignans are characterised by CFs at m/z 415 [C_24_H_31_O_6_]^+^, m/z 401 [C_23_H_29_O_6_]^+^, m/z 331 [C_18_H_19_O_6_]^+^, m/z 330 [C_18_H_18_O_6_]^+^. Combined with the CFs and NLs of other subtypes, this type of compound could be rapidly determined among the many components. The biphenyl cyclooctene lignans are divided into four categories according to their structure and different connection groups. There are type 1 biphenyl cyclooctene lignans with closed loops (without OH), type 2 biphenyl cyclooctene lignans (with OH), type 3 biphenyl cyclooctene lignans (with OH and benzoyl), type 4 biphenyl cyclooctene lignans (with OH and angeloyl or tigloyl moieties). The loss of neutral molecules occurs through energy collisions. For example, type 3 biphenyl cyclooctene lignans (with OH and benzoyl) have a symmetrical structure with benzoyl groups, so the neutral loss of a benzoyl at 122 Da (C_6_H_5_COOH) are one of the major fragmentation pathways obtained by collision-induced dissociation. According to the loss of molecules including 18 Da (H_2_O), 122 Da (C_6_H_5_COOH), 30 Da (CH_2_O), the unknown component could be further identified as type 3 biphenyl cyclooctene lignans.

### 2.2. Lignans

Lignans are the main active ingredients in WWZ fruits. Based on the parent nucleus structure lignans were divided into biphenyl cyclooctene lignans and open-loop lignans. According to their fragmentions in multi-stage spectra, characteristic dissociation rules were obtained, which can be summarized as follows: firstly, the fragment ions 415 [C_24_H_31_O_6_]^+^, 401 [C_23_H_29_O_6_]^+^, 331 [C_18_H_19_O_6_]^+^, 330 [C_18_H_18_O_6_]^+^ 301 [C_17_H_17_O_5_]^+^ are can be identified as CFs of biphenyl cyclooctene lignans. On the contrary, open-loop lignans were identified according to CFs including 182 [C_9_H_10_O_4_]^+^, and 122 [C_7_H_6_O_2_]^+^. Additionally, biphenyl cyclooctene lignans were divided into four classes consisting of type 1 biphenyl cyclooctene lignans with closed loop (without OH), type 2 biphenyl cyclooctene lignans (with OH), type 3 biphenyl cyclooctene lignans (with OH and benzoyl) and type 4 biphenyl cyclooctene lignans (with OH and angeloyl or tigloyl units) on the basis of their structure and different groups. NLs often reveal the information related to the categories of compounds and also play an important role in the rapid identification of compounds. Type 1 biphenyl cyclooctene lignans easily lose neutral fragments of *m*/*z* 70 Da (C_5_H_10_) and 56 Da (C_4_H_8_) to produce five membered rings or six membered rings formed from the eight membered ring. While type 1 of the parent nucleus is not connected with hydroxyl groups, based on the characteristic NLs at *m*/*z* 18 Da (H_2_O), three other classes and type 1 were distinguished. NLs at *m*/*z* 122 Da (C_6_H_5_COOH) and *m*/*z* 100 Da (C_4_H_7_COOH) are the key to distinguishing between type 2 and type 3, type 4. Additionally, type 3 and type 4 exhibited benzoyl and angeloyl or tigloyl structures; 122 Da (C_6_H_5_COOH) and 100 Da (C_4_H_7_COOH) peaks are characteristic NLs of type 3 and type 4 respectively. Lignans show adduct ions [M + Na]^+^ in positive mode; however, without signals in the negative ion mode [6,10,11,27,28,29,30,31,32,33]. Thus, substances in the WWZ extract were rapidly classified and identified using CFF combined with NLF.

**Figure 2:**
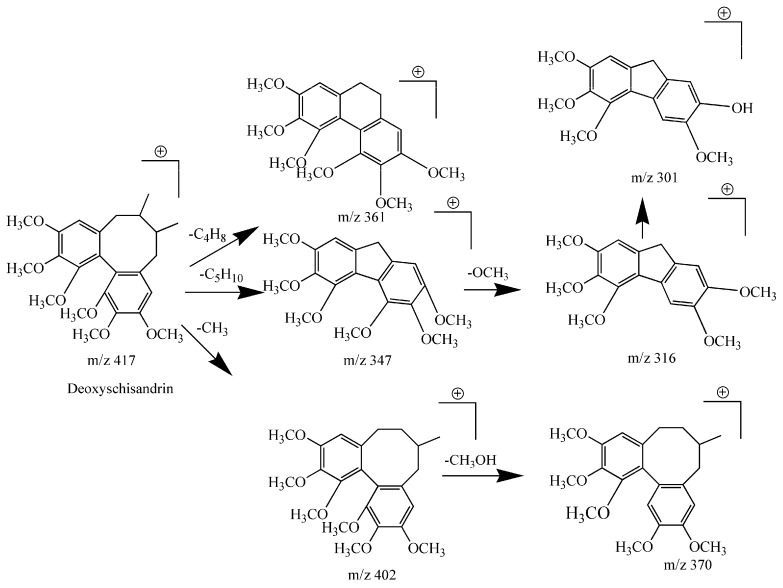
The proposed fragmentation pathway of deoxyschisandrin in positive ion mode.

Compound **1** had a retention time of 17.07 and formula of C_24_H_32_O_6._ In the positive ion mode, fragment ions at *m*/*z* 417 [M + H]^+^, *m*/*z* 439 [M + Na]^+^, 440 [M + H + Na]^+^, *m*/*z* 402 [M + H − CH_3_]^+^, *m*/*z* 347 [M + H − C_5_H_10_]^+^, *m*/*z* 361 [M + H − C_4_H_8_]^+^, *m*/*z* 370 [M + H − CH_3_ − CH_3_OH]^+^, *m*/*z* 316 [M + H − C_5_H_10_ − OCH_3_]^+^, *m*/*z* 301 [M + H − C_5_H_10_ − OCH_3_ − CH_3_]^+^ were obtained. According to the fragmentation rules, the fragment ion at *m*/*z* 301 [M + H − C_5_H_10_ − OCH_3_ − CH_3_]^+^ is a CFs that can be identified as corresponding to a biphenyl cyclooctene lignan. In addition, this compound presented *m*/*z* 417 [M + H]^+^, *m*/*z* 439 [M + Na]^+^, *m*/*z* 440 [M + H + Na]^+^ peaks and exhibited an NLs of 70 Da (C_5_H_10_) between the parent ion (*m*/*z* 417) and the fragment ion (*m*/*z* 347); this result confirmed the eight membered ring reaction; thus, this compound can be inferred as a type 1 biphenyl cyclooctene lignan. The fragment ion at *m*/*z* 402 [M + H − CH_3_]^+^was derived from the loss of one methyl (CH_3_) molecule. The fragment ion at *m*/*z* 370 [M + H − CH_3_ − CH_3_OH]^+^ displayed loss of a molecule of CH_3_OH on the basis of *m*/*z* 402 [M + H − CH_3_]^+^, while *m*/*z* 316 [M + H − C_5_H_10_ − OCH_3_]^+^ corresponds to the loss of a methoxyl group on the basis of *m*/*z* 347 [M + H − C_5_H_10_]^+^. Taken together, this information allows the compound to be identified as deoxyschisandrin [10,27,28] (for more details, see Table 2). The specific fragmentation pathways for deoxyschisandrin are shown in Figure 2.

**Figure 3:**
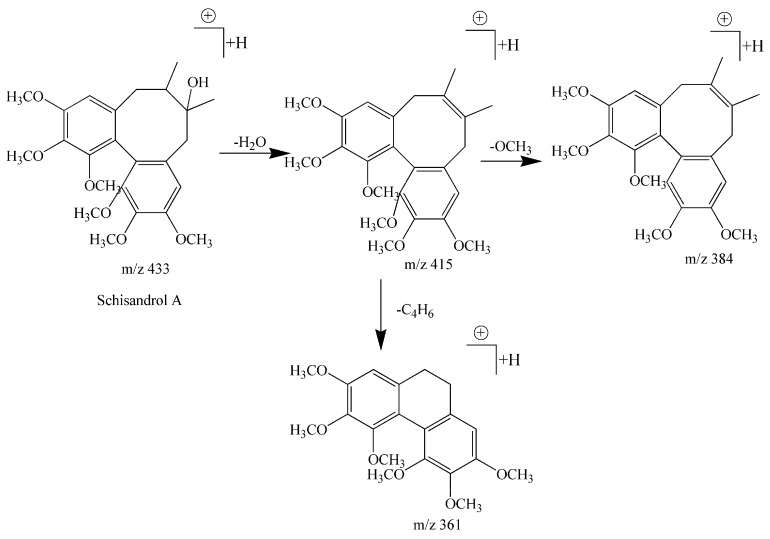
The fragmentation pathways of schisandrol A in positive ion mode.

Compound **5** had a retention time of 10.35, formula of C_24_H_32_O_7_, and showed fragment ions at *m*/*z* 433 [M + H]^+^, *m*/*z* 455 [M + Na]^+^, *m*/*z* 415 [M + H − H_2_O]^+^, *m*/*z* 384 [M + H − H_2_O − OCH_3_]^+^, *m*/*z* 361 [M + H − H_2_O − C_4_H_6_]^+^. The fragment ion at *m*/*z* 415 [M + H − H_2_O]^+^ exhibited an neutral loss of 18 Da for a H_2_O molecule, while the occurrence of the *m*/*z* 361 [M + H − H_2_O − C_4_H_6_]^+^ peak indicated a loss of 54 Da (C_4_H_6_) based on the fragment ion at *m*/*z* 415. According to the NL rules, compound **5** can be inferred as a type 2 biphenyl cyclooctene lignans (with OH). Additionally, a fragmentation at *m*/*z* 384 [M − H_2_O − OCH_3_]^+^ was obtained by the fragment ion *m*/*z* 415 [M + H − H_2_O]^+^ to lose a methoxyl group (OCH_3_). This fragmentation information combined with reference data allowed the compound to be identified as schisandrol A [11,29,30], and its cleavage pathways are shown in Figure 3.

Compound **8** had a retention times of 14.90 and the formula C_30_H_32_O_9._ In our experiments, we obtained fragment ions at *m*/*z* 537 [M + H]^+^, *m*/*z* 560 [M + H + Na]^+^, *m*/*z* 519 [M + H − H_2_O]^+^, *m*/*z* 415 [M + H − C_6_H_5_COOH]^+^, and *m*/*z* 385 [M + H − C_6_H_5_COOH − CH_2_O]^+^. After reviewing the literature and some reference experiments, the cleavage pathway suggested a type 3 biphenyl cyclooctene lignan which easily loses 18 Da (H_2_O), 122 Da (C_6_H_5_COOH), 30 Da (CH_2_O). Additionally the fragment ions at 519 [M + H − H_2_O]^+^, *m*/*z* 415 [M + H − C_6_H_5_COOH]^+^, *m*/*z* 385 [M + H − C_6_H_5_COOH − CH_2_O]^+^ confirmed the compound was a type 3 biphenyl cyclooctene lignan.

**Figure 4:**
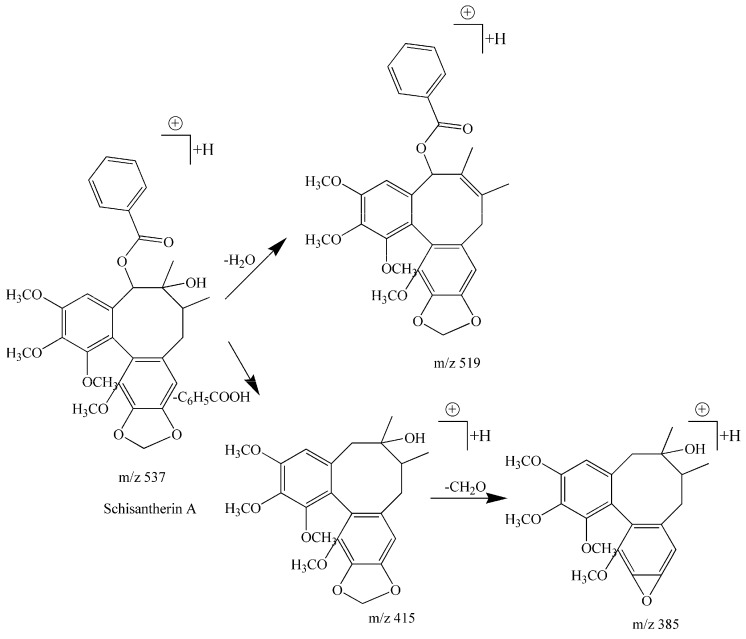
The specific fragmentation process of schisantherin A in positive ion mode.

The presence of the 415 [M + H − C_6_H_5_COOH]^+^ ion indicated a benzoyloxy group in the structure. The fragmention at *m*/*z* 537 [M + H]^+^ was a molecular ion, which resulted in a fragment ion at *m*/*z* 560 [M + H + Na]^+^. Therefore, comparing the fragment ions rules from the literature [10,29], compound **8** was determined to be schisantherin A. The specific fragmentation pathways of schisantherin A are shown in Figure 4.

### 2.3. Triterpenoids

Triterpenoids, according to their chemical structure, can be divided into three types: lanostane-type triterpenoids, cycloartane-type triterpenoids, and schisanra-type triterpenoids. Firstly, lanostane-type triterpenoids usually have carboxyl groups, and easily lose 46 Da (HCOOH) and 45 Da (HCOO^−^) molecules. Secondly, based on the relative abundance of the fragments at *m*/*z* 312 [C_22_H_32_O]^+^, 271 [C_19_H_27_O]^+^ in the MS data, the components which belong to cycloartane-type triterpenoids can be identified. Finally, schisanra-type triterpenoids differ from the other two types, and could be identified by their typical NLs, including 60 Da (CH_2_C(OH)_2_) and 74 Da (CH_3_CH=C(OH)_2_) that appeared in the spectra. Thus, based on the CFs and NLs we could rapidly determine the components which belong to the different types of triterpenoids [33,34,35].

**Figure 5:**
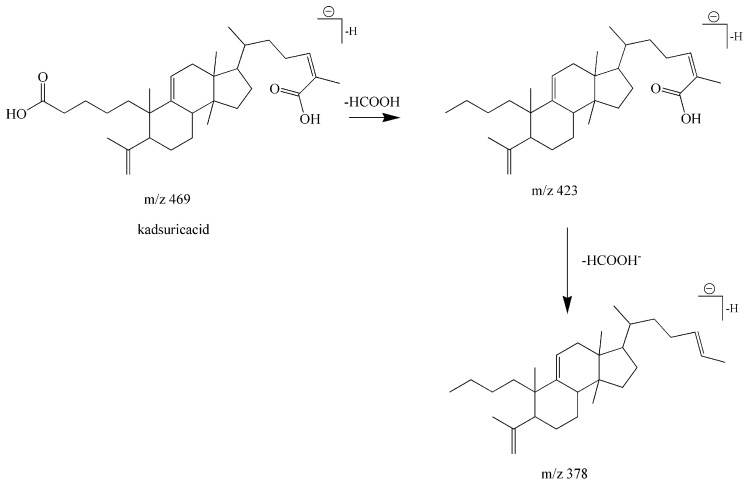
The specific fragmentation process of kadsuricacid in negative ion mode.

Compound **19** presented a retention time of 19.56 min and a formula of C_30_H_46_O_4_. Several main fragment ions at *m*/*z* 469 [M − H]^−^, *m*/*z* 423 [M − H − HCOOH]^−^, *m*/*z* 378 [M − H − HCOOH − HCOO]^−^ were observed in the multi-stage spectra. The characteristic dissociation rules of lanostane-type triterpenoids showed the loss of neutral molecules of 46 Da (HCOOH), 45 Da (HCOO^−^) dominate in the multi-stage fragmentations, resulting in the formation of 423 [M − H − HCOOH]^−^, *m*/*z* 378 [M − H − HCOOH − HCOO]^−^ ions, respectively. Additionally, the protonated molecular ion 469 [M − H]^−^ was observed. These results are consistent with the fragmentation pathway of kadsuricacid [35]. A proposed mechanistic pathway for fragments formed in negative ion mode is shown in Figure 5.

**Figure 6:**
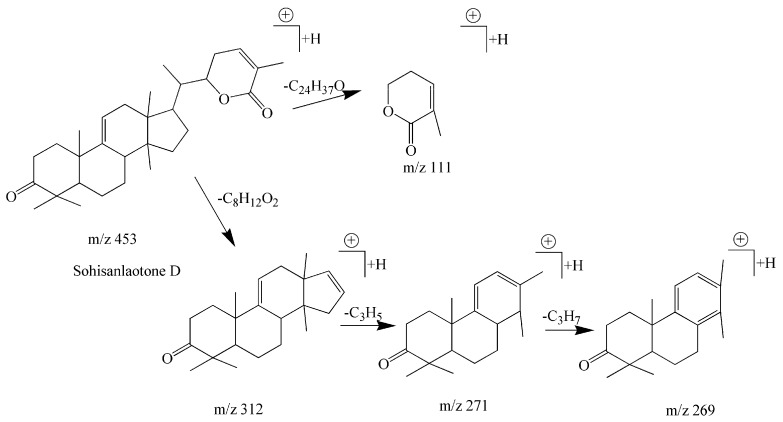
A fragmentation pathway of sohisanlaotone D in positive ion mode.

Compound **15** possessed a retention time of 20.48 min and a formula of C_30_H_44_O_3._ In the positive ion mode, it showed several fragments at *m*/*z* 453 [M + H]^+^, *m*/*z* 312 [M − C_8_H_12_O_2_]^+^, *m*/*z* 271 [M − C_8_H_12_O_2_ − C_3_H_5_]^+^, *m*/*z* 269 [M − C_8_H_12_O_2_ − C_3_H_7_]^+^, *m*/*z* 111 [C_6_H_7_O_2_]^+^. The ions at *m*/*z* 312 [C_22_H_32_O]^+^ and *m*/*z* 271 [C_19_H_27_O]^+^ as CFs suggested this compound belonged to the cycloartane-type triterpenoids, and the fragment ion at *m*/*z* 111 [C_6_H_7_O_2_]^+^ indicated loss of a benzoyl fragment from the parent nucleus. The fragment at *m*/*z* 453 [M+H]^+^ is the protonated molecular ion. Thus, compound **15** was determined as sohisanlaotone D by combining the fragmentation rules with literature data [35]. A pathway for the fragments formed in the MS is shown in Figure 6.

**Figure 7:**
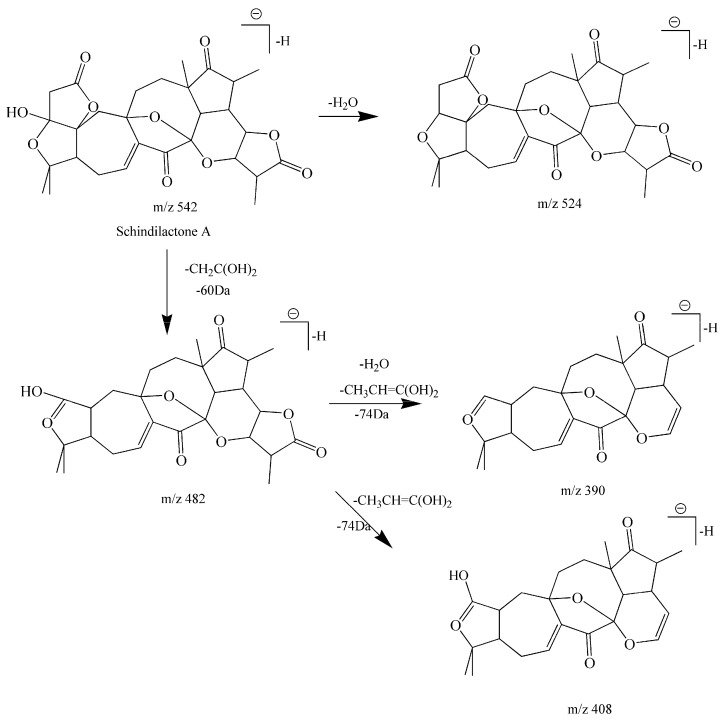
The proposed fragmentation pathway of schisantherin A.

Compound **24** was eluted at 8.79 min and its molecular formula was C_29_H_35_O_10._ The fragments included peaks at *m*/*z* 542[M − H]^−^, *m*/*z* 524 [M − H − H_2_O]^−^, *m*/*z* 482 [M − H − CH_2_C(OH)_2_]^−^, *m*/*z* 408 [M − H − CH_2_C(OH)_2_ − CH_3_CH=C(OH)]^−^, *m*/*z* 390 [M − H − H_2_O − CH_2_C(OH)_2_ − CH_3_CH=C(OH)]^−^ obtained in negative ion mode, and 60 Da (CH_2_C(OH)_2_) and 74 Da (CH_3_CH=C(OH)_2_) are characteristic NLs of schisanra-type triterpenoids according to the consulted literature information and mass fragmentation patterns (NLF). The ions at *m*/*z* 408 [M − H − CH_2_C(OH)_2_ − CH_3_CH=C(OH)]^−^, *m*/*z* 390 [M − H − H_2_O − CH_2_C(OH)_2_ − CH_3_CH=C(OH)]^−^ further support the identification of this type of triterpenoid. The fragmentation at *m*/*z* 524 [M − H − H_2_O]^−^ was obtained from the fragment at *m*/*z* 542 [M − H]^−^ by loss of a molecule of H_2_O. These results are in conformity with the CF and NL rules shown in Table 1. Therefore, compound **24** was determined as schindilactone A [33]. The proposed fragmentation pathways of schisantherin A are shown in Figure 7.

### 2.4. Fatty Acids

Fatty acids are divided into saturated fatty acids and unsaturated fatty acids. CFs at *m*/*z* 74 [C_5_H_14_]^+^ of saturated fatty acids produced by Mclafferty rearrangements in the mass spectrometer were observed. Unsaturated fatty acids undergo fragmentations through hydrogen transfer, gamma, and alpha cleavage that produce CFs of 55 [C_4_H_7_]^+^, 67 [C_5_H_7_]^+^, 79 [C_6_H_7_]^+^ or 54 [C_4_H_6_]^−^, 66 [C_5_H_6_]^−^, 78 [C_6_H_6_]^−^. As a result, we can quickly identify the different types of fatty acids, such as compound **26**, for which *m*/*z* 66 [C_5_H_6_]^−^, *m*/*z* 261 [M − H − H_2_O]^−^, *m*/*z* 279 [M−H]^−^ peaks were obtained. According to the fragmentation rules and other ion information, the compound showing an ion of [M − H]^−^ at *m*/*z* 279 corresponding to the molecular formula C_18_H_32_O_2_, can be inferred as 9,12-linoleic acid [36]. For more details, see Table 3.

### 2.5. Organic Acids

Organic acids play a major role in antibiotics [8]. According to our literature review this kind of compound strongly loses acid groups [37], besides, organic acids easily produce H_2_O and CO_2_ groups in negative ion mode. Thus, this kind of compound was identified on the basis of the parention fragments [M+H]^−^ combined with neutral losses. For example, compound **28**, ions at *m*/*z* 191 [M − H]^−^, *m*/*z* 146 [M − HCOOH]^−^, *m*/*z* 147 [M − H − CO_2_]^−^, *m*/*z* 129 [M − H − CO_2_−H_2_O]^−^, *m*/*z* 85 [M − H − CO_2_ - CO_2_ − H_2_O]^–^ were obtained in the negative ion mode, and ones at *m*/*z* 146 [M − HCOOH]^−^, *m*/*z* 147 [M − H − CO_2_]^−^, corresponding to the NLs of 44 Da (CO_2_), 18 Da (H_2_O) and the parention fragmentation *m*/*z* 191 [M − H]^-^ indicated citric acid [37,38], which formula is C_6_H_8_O_7_, shown in Table 3.

## 3. Materials and Methods

### 3.1. Sample Preparation

The dried WWZ fruits were pulverized and then the coarse powder (10 g) was accurately weighed and extracted twice with four times the amount of 85% ethanol (made up of 34 mL of ethanol and 6 mL of water). Each reflux time lasted 3 h at room temperature (about 25 °C). The combination of the two extracts was filtered, and the filtrate was subsequently passed through a 0.22 µm membrane and then 2 µL of the sample was injected into the UPLC-Q-TOF/MS for constituent analysis [28,39].

### 3.2. UPLC and MS Conditions

UPLC was performed using a Waters Acquity UPLC system (Waters, Milford, MA, USA), which consisted of a quaternary pump, an autosampler, a DAD detector and a column compartment. UPLC separation was achieved on Waters ACQUITY UPLC BEH C_18_ column (100 mm × 2.1 mm, 1.7 μm particle size). The mobile phase composed of (**A**) 0.1% formic acid in water and (**B**) acetonitrile containing 0.1% formic acid. The gradient elution program employed was as follows: 0–5min, 20–30%**B**; 5–15min, 30–60%**B**; 15–20min, 60–90%**B**; 20–25min, 90–90%**B**; 25–30min, 90–20%**B**; 30–35min, 20–20%**B**. The flow rate was 0.4 mL/min. The column and autosampler were maintained at 35 °C [24,25,28,39]. The UPLC system was coupled to a Q-TOF-MS instrument equipped with electrospray ionization (ESI) in positive and negative ion modes. Ultra-high purity helium (He) was used as the collision gas and high-purity nitrogen (N_2_) was used as nebulizing gas. The range of the data acquisition was 50 to 1000 Da. Other conditions of ESI source were as follows: capillary voltage, 3.5 kV; collision energy, 20–40 eV, drying gas temperature, 325 °C; desolvation gas flow rate, 600 L h^−1^; and nebulizing gas pressure, 350 psi. The Leu-Enkephalin ions at *m*/*z* 556.2771 and 554.2615 were used to calibrate the mass accuracy [24,25,28,39].

## 4. Conclusions

In this study, based on a powerful integrated approach that UPLC-Q-TOF/MS combined with data post-processing technology, 30 compounds were screened successfully (for further information, see Table 2 and Table 3). The structures of compounds were confirmed by using their MS fragments and by comparison with reference standards and corresponding reference data on the basis of the typical cleavage pathways of four chemical constituent classes in WWZ. In addition to the main component lignans found in WWZ, it also contains triterpenoids, organic acids and fatty acids. The method described in this report has high resolution and sensitivity that reduces difficulty of identification of complicated and diverse components in WWZ fruits, and laid the foundation for study on pharmacological and pharmacokinetics in WWZ fruits. To some extent, it made up for the deficiency of the existing analytic methods for TCMs. Furthermore, the novel strategy was a powerful tool for the systematic screening and identification of quality control and chemical analysis of TCM, and promoted the development of TCM.

## Figures and Tables

**Table 1:** Characteristic fragments and neutral loss of different chemical constituents in WWZ extract.

Classification	Subclass	Neutral Loss	Characteristic Fragments
Lignans	Type 1 biphenyl cyclooctene lignans (without OH)	70 Da (C_5_H_10_) 56 Da (C_4_H_8_)	415 [C_24_H_31_O_6_]^+^ 401 [C_23_H_29_O_6_]^+^ 331 [C_18_H_19_O_6_]^+^ 330 [C_18_H_18_O_6_]^+^ 301 [C_17_H_17_O_5_]^+^
	Type 2 biphenyl cyclooctene lignans (with OH)	18 Da (H_2_O) 54 Da (C_4_H_6_)	
	Type 3 biphenyl cyclooctene lignans (with OH and benzoyl),	18 Da (H_2_O) 122 Da (C_6_H_5_COOH) 30 Da (CH_2_O)	
	Type 4 biphenyl cyclooctene lignans (with OH and angeloyl or tigloyl)	18 Da (H_2_O) 100 Da (C_4_H_7_COOH) 30 Da (CH_2_O)	
	Open-loop lignans		182 [C_9_H_10_O_4_]^+^ 122 [C_7_H_6_O_2_]^+^
Triterpenoids	Lanostane-type	46 Da (HCOOH) 45 Da (HCOO^−^)	
	Cycloartane-type		312 [C_22_H_32_O]^+^ 271 [C_19_H_27_O]^+^
	Schisanra-type	60 Da (CH_2_C(OH)_2_) 74 Da (CH_3_CH=C(OH)_2_)	
Fatty acids			55 [C_4_H_7_]^+^ or 54[C_4_H_6_]^−^ 67 [C_5_H_7_]^+^ or 66[C_4_H_6_]^−^ 79 [C_6_H_7_]^+^ or 78[C_4_H_6_]^−^
Organic acids		44 Da (CO_2_) 18 Da (H_2_O)	

**Table 2:** Identification of the chemical constituents of the WWZ extract by using UPLC-Q-TOF/MS in positive ion modes.

No.	Classification	Name	Formula	*m*/*z* [M + H]^+^	Time (min)	Experimental * m*/*z* [M + H]^+^	ppm	Fragment Ions	Ref
**1**	Type 1 biphenyl cyclooctene lignans (without OH)	Deoxyschisandrin	C_24_H_32_O_6_	417.2277	17.07	417.2289	2.88	417 [M + H]^+^ 439 [M + Na]^+^ 440 [M + H + Na]^+^ 402 [M + H − CH_3_]^+^ 347 [M + H − C_5_H_10_]^+^ 370 [M + H − CH_3_ − CH_3_OH]^+^ 316 [M + H − C_5_H_10_ − OCH_3_]^+^ 301 [M + H − C_5_H_10_ − OCH_3_ − CH_3_]^+^ 361 [M+H − C_4_H_8_]^+^	[10,27,28]
**2**	Type 1 biphenyl cyclooctene lignans (without OH)	SchisandrinB	C_23_H_28_O_6_	401.1964	17.97	401.1962	0.50	401 [M + H]^+^ 423 [M + Na]^+^ 386 [M + H − CH_3_]^+^ 331 [M + H − C_5_H_10_]^+^ 301 [M + H − C_5_H_10_− OCH_3_]^+^ 371 [M + H − CH_3_− CH_3_]^+^	[10,11,28]
**3**	Type 1 biphenyl cyclooctene lignans (without OH)	Schisandrin C	C_22_H_24_O_6_	385.1651	18.41	385.1642	2.08	385 [M + H]^+^ 407 [M + Na]^+^ 370 [M + H − CH_3_]^+^ 315 [M + H − C_5_H_10_]^+^ 300 [M + H − C_5_H_10_ − CH_3_]^+^	[10]
**4**	Other lignans	Gomisin J	C_22_H_28_O_6_	389.1964	11.43	389.1953	2.83	389 [M + H]^+^ 411 [M + Na]^+^ 319 [M + H − C_5_H_10_]^+^ 342 [M + H − CH_3_OH − CH_3_]^+^ 358 [M + H − OCH_3_]^+^ 374 [M + H − CH_3_]^+^	[10]
**5**	Type 2 biphenyl cyclooctene lignans (with OH)	Schisandrol A	C_24_H_32_O_7_	433.2226	10.35	433.2226	0.00	433 [M + H]^+^ 455 [M + Na]^+^ 415 [M + H − H_2_O]^+^ 384 [M + H − H_2_O − OCH_3_]^+^ 361 [M + H − H_2_O − C_4_H_6_]^+^	[11,29,30]
**6**	Type 2 biphenyl cyclooctene lignans (with OH)	Schisandrol B	C_23_H_28_O_7_	417.1913	11.58	417.1889	5.75	417 [M + H]^+^ 439 [M + Na]^+^ 399 [M + H − H_2_O]^+^ 345 [M + H − H_2_O − C_4_H_6_]^+^ 367 [M + H − H_2_O − OCH_3_]^+^	[10]
**7**	Type 2 biphenyl cyclooctene lignans (with OH)	Schisanhenol	C_23_H_30_O_6_	403.212	14.50	403.2109	2.73	403 [M + H]^+^ 425 [M + Na]^+^ 385 [M + H − H_2_O]^+^ 331 [M + H − H_2_O − C_4_H_6_]^+^ 354 [M + H − H_2_O − OCH_3_]^+^	[10]
**8**	Type 3 biphenyl cyclooctene lignans (with OH and benzoyl)	Schisantherin A	C_30_H_32_O_9_	537.2124	14.90	537.2104	3.72	537 [M + H]^+^ 560 [M + H + Na]^+^ 519 [M + H − H_2_O]^+^ 415 [M + H − C_6_H_5_COOH]^+^ 385 [M + H − C_6_H_5_COOH − CH_2_O]^+^ 371 [M + H − C_6_H_5_COOH − C_2_H_4_O]^+^	[10,11,29]
**9**	Type 3 biphenyl cyclooctene lignans(with OHand benzoyl)	Gomisin G	C_30_H_32_O_9_	537.2124	14.55	537.2114	1.86	537 [M + H]^+^ 559 [M + Na]^+^ 519 [M + H − H_2_O]^+^ 415 [M + H − C_6_H_5_COOH]^+^ 385 [M + H − C_6_H_5_COOH − CH_2_O]^+^ 371 [M + H − C_6_H_5_COOH − C_2_H_4_O]^+^ 340 [M + H − C_6_H_5_COOH − C_2_H_4_O − OCH_3_]^+^	[10]
**10**	Type 3 biphenyl cyclooctene lignans (with OH and benzoyl)	Schisantherin D	C_29_H_28_O_9_	521.1811	15.09	521.1818	1.34	521 [M + H]^+^ 543 [M + Na]^+^ 559 [M + K]^+^ 399 [M + H − C_6_H_5_COOH]^+^ 369 [M + H − C_6_H_5_COOH − CH_2_O]^+^	[10]
**11**	Type 3 biphenyl cyclooctene lignans (with OH and benzoyl)	Benzoyl gomisin O	C_30_H_32_O_8_	521.2175	18.63	521.2138	7.10	521 [M + H]^+^ 543 [M + Na]^+^ 399 [M + H − C_6_H_5_COOH]^+^ 369 [M + H − C_6_H_5_COOH − CH_2_O]^+^ 384 [M + H − C_6_H_5_COOH − CH_3_]^+^	[10]
**12**	Type 3 biphenyl cyclooctene lignans (with OH and benzoyl)	Benzoyl iso- gomisin O	C_30_H_32_O_8_	521.2175	18.62	521.2128	9.01	521 [M + H]^+^ 399 [M + H − C_6_H_5_COOH]^+^ 384 [M + H − C_6_H_5_COOH − CH_3_]^+^	[33]
**13**	Other lignans	Gomisin K1	C_23_H_31_O_6_	404.2199	15..36	404.2158	10.14	403 [M]^+^	[27]
**14**	Other lignans	Pregomisin	C_22_H_31_O_6_	387.1807	16.39	387.1801	1.55	391 [M + H]^+^ 361 [M + H − CH_2_O]^+^ 331 [M + H − 2CH_2_O]^+^ 373 [M + H − H_2_O]^+^ 358 [M + H − H_2_O − CH_3_]^+^	[10]
**15**	Cycloartane-type triterpenoids	Sohisanlaotone D	C_30_H_44_O_3_	453.3368	20.48	453.3349	4.19	453 [M + H]^+^ 312 [M − C_8_H_12_O_2_]^+^ 271 [M − C_8_H_12_O_2_ − C_3_H_5_]^+^ 269 [M − C_8_H_12_O_2_ − C_3_H_7_]^+^ 111 [M − C_24_H_37_O]^+^	[35]
**16**	Fatty Acids	Eicosapentaenoic Acid	C_20_H_30_O_2_	303.2324	20.33	303.2294	9.89	303 [M + H]^+^ 79 [C_6_H_7_]^+^	[36]
**17**	Lanostane-type triterpenoids	Micranoic acid B	C_22_H_32_O_3_	343.2273	20.03	345.2415	4.06	345 [M + H]^+^ 299 [M + H − HCOOH]^+^	[35]

**Table 3:** Identification of the chemical constituents of the WWZ extract by using UPLC-Q-TOF/MS in negative ion modes.

No.	Classification	Name	Formula	*m*/*z* [M − H]^−^	Time (min)	Experimental *m*/*z* [M − H]^−^	ppm	Fragment Ions	Ref.
**18**	Open-loop lignans	3′,4′-Dimethoxybenzoicacid-(3′′,4′′-dimethoxyphenyl)-2-methyl-3-oxobutyl ester	C_22_H_26_O_7_	401.1601	12.68	401.1614	3.24	401 [M − H]^−^ 182 [C_9_H_10_O_4_]^−^	[32]
**19**	Lanostane-type triterpenoids	Kadsuricacid	C_30_H_46_O_4_	469.3318	19.56	469.3318	0.00	469 [M − H]^−^ 423 [M − H − HCOOH]^−^ 378 [M − H − HCOOH − HCOO]^−^	[35]
**20**	Lanostane-type triterpenoids	Kadsuricacid 3-methyl dster	C_31_H_48_O_4_	483.3475	23.02	483.3483	1.66	483 [M − H]^−^ 452 [M − H − OCH_3_]^−^ 407 [M − H − OCH_3_ − HCOO]^−^	[34]
**21**	Cycloartane-type triterpenoids	Ganwuweizic acid	C_30_H_46_O_3_	453.3369	21.77	453.3368	0.22	453[M − H]^−^ 435 [M − H − H_2_O]^−^	[35]
**22**	Schisanra-type triterpenoids	Henridilactone A	C_29_H_35_O_10_	542.2152	8.91	542.2133	3.50	542 [M − H]^−^524 [M – H − H_2_O]^−^464 [M − H − H_2_O − CH_2_C(OH)_2_]^−^390 [M – H − H_2_O − CH_2_C(OH)_2_ − CH_3_CH=C(OH)]^−^	[33]
**23**	Schisanra-type triterpenoids	Lancifodilactone D	C_29_H_35_O_9_	526.2203	9.50	526.2167	6.84	526 [M − H]^−^ 466 [M – H − CH_2_C(OH)_2_]^−^ 448 [M − H − CH_2_C(OH)_2_ − H_2_O]^−^	[33]
**24**	Schisanra-type triterpenoids	Schindilactone A	C_29_H_35_O_10_	542.2152	8.79	542.2139	2.40	542 [M − H]^−^ 524 [M – H − H_2_O]^−^ 482 [M – H − CH_2_C(OH)_2_]^−^ 408 [M − H − CH_2_C(OH)_2_ − CH_3_CH=C(OH]^−^ 390 [M − H− H_2_O − CH_2_C(OH)_2_ − CH_3_CH=C(OH]^−^	[33]
**25**	Other terpenoids	Lancifodilactone c	C_29_H_36_O_10_	543.223	5.07	543.2225	0.92	543 [M − H]^−^ 525 [M − H − H_2_O]^−^ 445[M − H − C_4_H_7_O_2_]^−^ 499 [M − H − COO]^−^ 481 [M − H − H_2_O − COO]^−^	[34]
**26**	Fatty Acids	9,12-Linoleic acid	C_18_H_32_O_2_	279.2324	20.33	279.2329	1.79	66 [C_5_H_6_]^−^ 261 [M − H − H_2_O]^−^ 279 [M − H]^−^	[36]
**27**	Fatty Acids	α-Linolenic acid	C_18_H_30_O_2_	277.2168	19.31	277.2173	1.80	277 [M − H]^−^ 78 [C_6_H_6_]^−^	[36]
**28**	Organic acids	Citric acid	C_6_H_8_O_7_	191.0192	0.78	191.0199	3.66	191 [M − H]^−^ 146 [M − HCOOH]^−^ 147 [M − H − CO_2_]^−^ 129 [M − H − CO_2_ − H_2_O]^−^ 85 [M − H − CO_2_ − CO_2_ − H_2_O]^−^	[37,38]
**29**	Organic acids	6-methyl citrate	C_7_H_10_O_7_	205.0349	0.988	205.0353	1.95	205 [M − H]^−^ 161 [M − H − CO_2_]^−^ 174 [M − H − OCH_3_]^−^	[37]
**30**	Organic acids	Dimethyl citrate	C_8_H_12_O_7_	219.0505	1.08	219.0508	1.37	219 [M − H]^−^ 175 [M − H − CO_2_]^−^ 188 [M − H − OCH_3_]^−^	[37]
